# Supplementary material for: MetaRibo-Seq measures translation in microbiomes
Source: Nat Commun. 2020 Jun 29;11:3268. doi: 10.1038/s41467-020-17081-z (PMC7324362; doi:10.1038/s41467-020-17081-z)
Supplement: Supplementary file 10 — Supplementary Data 7 [file 41467_2020_17081_MOESM10_ESM.zip › File2/Confidence_High_Taxonomy/241262_out.krona.html]

Javascript must be enabled to view this page.

members
magnitude
magnitudeUnassigned
count
unassigned
taxon
rank

241262\_out

84

84
2
superkingdom

phylum
84
1239

class
84
186801

order
84
186802

73
186803
family

species
39491
73

SRS012849\_contig\_number\_35509SRS013940\_contig\_number\_7337SRS014287\_contig\_number\_1277SRS014313\_contig\_number\_4733SRS014459\_contig\_number\_28225SRS014613\_contig\_number\_22275SRS014683\_contig\_number\_18846SRS014736\_contig\_number\_291SRS014855\_contig\_number\_10633SRS014948\_contig\_number\_20925SRS015065\_contig\_number\_20896SRS015579\_contig\_number\_14309SRS015794\_contig\_number\_10820SRS018313\_contig\_number\_7641SRS019030\_contig\_number\_16375SRS019267\_contig\_number\_1447SRS019968\_contig\_number\_17786SRS020394\_contig\_number\_11923SRS022137\_contig\_number\_22124SRS023526\_contig\_number\_29805SRS023715\_contig\_number\_22200SRS024075\_contig\_number\_15217SRS024132\_contig\_number\_5008SRS044535\_contig\_number\_24500SRS045244\_contig\_number\_7972SRS045826\_contig\_number\_24781SRS046369\_contig\_number\_16201SRS046717\_contig\_number\_3838SRS047433\_contig\_number\_14680SRS048060\_contig\_number\_13385SRS048164\_contig\_number\_30062SRS049446\_contig\_number\_11459SRS054590\_contig\_number\_1246SRS055017\_contig\_number\_29195SRS055982\_contig\_number\_19275SRS056519\_contig\_number\_12105SRS058723\_contig\_number\_6586SRS062427\_contig\_number\_9776SRS062654\_contig\_number\_27008SRS064557\_contig\_number\_16862SRS065397\_contig\_number\_9695SRS074964\_contig\_number\_6704SRS075773\_contig\_number\_27763SRS077335\_contig\_number\_9598SRS077502\_contig\_number\_5997SRS098717\_contig\_number\_23736SRS1041090\_contig\_number\_5862SRS1041091\_contig\_number\_19049SRS1041145\_contig\_number\_26747SRS104327\_contig\_number\_3287SRS104485\_contig\_number\_4910SRS142712\_contig\_number\_4926SRS143070\_contig\_number\_12631SRS143342\_contig\_number\_10936SRS143372\_contig\_number\_8338SRS143466\_contig\_number\_30078SRS143523\_contig\_number\_24513SRS143991\_contig\_number\_30265SRS144537\_contig\_number\_33091SRS144714\_contig\_number\_19160SRS146764\_contig\_number\_31910SRS146813\_contig\_number\_15641SRS147271\_contig\_number\_16716SRS147346\_contig\_number\_50653SRS148196\_contig\_number\_22092SRS148253\_contig\_number\_11049SRS149075\_contig\_number\_19546SRS149325\_contig\_number\_15786SRS893230\_contig\_number\_176SRS893231\_contig\_number\_1830SRS893341\_contig\_number\_16119SRS893369\_contig\_number\_11341SRS893373\_contig\_number\_24791

8
186806
family

1730
8
genus

species
142586
8

SRS011239\_contig\_number\_contig-100\_2137.153741SRS014923\_contig\_number\_31364SRS017521\_contig\_number\_45895SRS017701\_contig\_number\_21560SRS018656\_contig\_number\_9499SRS052697\_contig\_number\_25125SRS145497\_contig\_number\_20721SRS971275\_contig\_number\_33759

family
541000
1

1
216851
genus


SRS893170\_contig\_number\_10646
1
853
species

2
31979
family

1649459
1
genus

species

SRS146888\_contig\_number\_13422
1
154046

1
1485
genus


SRS104311\_contig\_number\_33260
1
1262789
species
